# Comparison of machine learning methods with logistic regression analysis in creating predictive models for risk of critical in-hospital events in COVID-19 patients on hospital admission

**DOI:** 10.1186/s12911-022-02057-4

**Published:** 2022-11-28

**Authors:** Aaron W. Sievering, Peter Wohlmuth, Nele Geßler, Melanie A. Gunawardene, Klaus Herrlinger, Berthold Bein, Dirk Arnold, Martin Bergmann, Lorenz Nowak, Christian Gloeckner, Ina Koch, Martin Bachmann, Christoph U. Herborn, Axel Stang

**Affiliations:** 1 Semmelweis University, Asklepios Campus Hamburg, Budapest, Hungary; 2 Asklepios Proresearch, Research Institute, Hamburg, Germany; 3 Department of Cardiology and Intensive Care Medicine, Asklepios Hospital St. Georg, Hamburg, Germany; 4 Department of Internal Medicine, Asklepios Hospital Nord-Heidberg, Hamburg, Germany; 5 Asklepios Tumorzentrum, Hamburg, Germany; 6 Department of Anesthesiology and Intensive Care Medicine, Asklepios Hospital St. Georg, Hamburg, Germany; 7 Department of Hematology, Oncology, Palliative Care and Rheumatology, Asklepios Hospital Altona, Hamburg, Germany; 8 Department of Internal Medicine, Cardiology, and Pneumology, Asklepios Hospital Wandsbek, Hamburg, Germany; 9 Department of Intensive Care and Ventilation Medicine, Asklepios Hospital München-Gauting, Gauting, Germany; 10 Department of Internal Medicine, Asklepios Hospital Oberviechtach, Oberviechtach, Germany; 11 Biobank for Pulmonary Diseases, Asklepios Hospital München-Gauting, Gauting, Germany; 12 Department of Intensive Care and Ventilatory Medicine, Asklepios Hospital Harburg, Hamburg, Germany; 13 Asklepios Hospitals GmbH & Co. KGaA, Hamburg, Germany; 14 Department of Hematology, Oncology and Palliative Care Medicine, Asklepios Hospital Barmbek, Rübenkamp 220, 22291 Hamburg, Germany

**Keywords:** COVID-19, Machine learning, Predictive models, Critical event prediction, Clinical decision-making

## Abstract

**Background:**

Machine learning (ML) algorithms have been trained to early predict critical in-hospital events from COVID-19 using patient data at admission, but little is known on how their performance compares with each other and/or with statistical logistic regression (LR). This prospective multicentre cohort study compares the performance of a LR and five ML models on the contribution of influencing predictors and predictor-to-event relationships on prediction model´s performance.

**Methods:**

We used 25 baseline variables of 490 COVID-19 patients admitted to 8 hospitals in Germany (March–November 2020) to develop and validate (75/25 random-split) 3 linear (L1 and L2 penalty, elastic net [EN]) and 2 non-linear (support vector machine [SVM] with radial kernel, random forest [RF]) ML approaches for predicting critical events defined by intensive care unit transfer, invasive ventilation and/or death (composite end-point: 181 patients). Models were compared for performance (area-under-the-receiver-operating characteristic-curve [AUC], Brier score) and predictor importance (performance-loss metrics, partial-dependence profiles).

**Results:**

Models performed close with a small benefit for LR (utilizing restricted cubic splines for non-linearity) and RF (AUC means: 0.763–0.731 [RF–L1]); Brier scores: 0.184–0.197 [LR–L1]). Top ranked predictor variables (consistently highest importance: C-reactive protein) were largely identical across models, except creatinine, which exhibited marginal (L1, L2, EN, SVM) or high/non-linear effects (LR, RF) on events.

**Conclusions:**

Although the LR and ML models analysed showed no strong differences in performance and the most influencing predictors for COVID-19-related event prediction, our results indicate a predictive benefit from taking account for non-linear predictor-to-event relationships and effects. Future efforts should focus on leveraging data-driven ML technologies from static towards dynamic modelling solutions that continuously learn and adapt to changes in data environments during the evolving pandemic.

*Trial registration number*: NCT04659187.

**Supplementary Information:**

The online version contains supplementary material available at 10.1186/s12911-022-02057-4.

## Background

The corona virus disease 2019 (COVID-19) pandemic, caused by the severe acute respiratory syndrome coronavirus 2 (SARS-CoV-2), puts emergency and intensive care units (ICU) worldwide into crisis [1]. Among COVID-19 patients, ~ 10% require hospitalisation and can rapidly deteriorate to life-threatening respiratory insufficiency, which carries a high mortality risk and requires immediate ICU and/or invasive mechanical ventilation (IMV) support [2, 3]. Prediction models that use clinical data on admission to stratify COVID-19 patients by risk for need of ICU support, IMV and/or in-hospital mortality could have a considerable clinical benefit for resource allocation and patient management [1, 4, 5]. However, a recent systematic review analysed 107 currently proposed predictive models to identify high-risk COVID-19 patients on hospital admission, concluding that all of these models are at high risk of bias mainly due to methodical issues, and none has been recommended for adoption into clinical practice [6].

Models to predict the risk of critical in-hospital events from COVID-19 were built from traditional logistic regression (LR) models and information-based criteria to data-driven machine learning (ML) algorithms, but few studies exist comparing the model development techniques [6–8]. However, traditional statistical LR models are based on probability distributions and focus on transparency of relationships between predictors and outcome, whereas ML approaches iteratively learn from examples and purely focus on prediction [9, 10]. Moreover, ML techniques differ in how they take account for non-linearities, interactions and correlations [10]. We therefore need more data on whether different models obtain different performance and/or predictors when applied to predict critical events based on real-world clinical data, which are inherently complex, multidimensional, heterogeneous, non-linear and noisy [9–11].

In this prospective observational multicentre cohort study, we applied a statistical LR model and five ML procedures to a similar clinical input dataset from COVID-19 patients on hospital admission. The aims were to compare the performance of the created models for prediction of critical in-hospital events from COVID-19, to assess overlaps and differences of the most influencing predictor variables between models, and to evaluate the contribution of predictor-to-event relationships and effects on model´s predictive performance.

## Methods

### Study design

The “CORONA Germany” - *Clinical Outcome and Risk in hospitalized COVID-19 patients* - study (ClinicalTrials.gov, NCT04659187) is a prospective, multicenter, observational, epidemiological cohort study. It is conducted in 45 hospitals across Germany that are all part of the same hospital network (Asklepios). Design and prior results of the CORONA-Germany-Study have been published previously [12]. This study analyses a predefined subcohort from 8 hospitals in Hamburg and Gauting which provided a comprehensive data set with detailed information on patient´s admission characteristics, in-hospital trajectories and outcome. All data are matched to and validated by the network´s quality management data base. An endpoint committee, provided by the networks research institute, reviewed all study endpoints. The ethics committees of the General Medical Councils (Aerztekammer) for the cities Hamburg and Munich approved the study and determined that this work was exempt from human subject´s research regulations since all information was collected on a fully anonymized basis. Therefore, the ethics committees waived the need for participant consent.

### Study cohort

We included 490 consecutive hospitalized patients who had laboratory-confirmed COVID-19 infection and were admitted to any of 8 hospitals in Hamburg and Gauting, Germany, between March 8th and September 15th 2020. A confirmed case of COVID-19 was defined by a positive polymerase chain reaction test of a nasopharyngeal swab. Each participant was followed up during hospital stay until discharge or death. Patient data were extracted anonymously from electronic patient charts of a hospital information technology network into pre-formatted data fields. The de-identified database contained data of demographic information, baseline vital parameters, baseline laboratory values, prior medication, pre-existing comorbidities, treatment processes, and survival data.

### Missing data and pre-selection of variables

After reviewing the existing literature regarding risk factors on COVID-19 outcome, we selected 44 baseline variables on admission as potential predictors of critical in-clinic events (thereafter referred to as input, Table 1). In the run-up of model building, 14 variables affected by informative missing and exhibiting ≥ 30% missing values were excluded. 14 variables were completely documented and 11 variables exhibit 8–134 missing values. Data were imputed using additive regression models and predictive mean matching [13]. The processed dataset comprises 25 variables, 12 continuous/discrete and 13 binary variables.

**Table 1:** Baseline characteristics of the COVID-19-infected study cohort on hospital admission (n = 490 study participants)

Characteristic	Non-missing cases	Statistics
*Demographics*		
Age (years)^a^	481	67 ± 18 72 [55, 81]
Gender (female: male)^a^	482	42 (203): 58 (279)
BMI (kg/m^2^)	81	28 ± 8 25 [23, 31]
*Symptoms*		
Cough^a^	453	58 (264)
Fever (> 38,5 °C)^a^	448	46 (205)
Dyspnea^a^	448	57 (256)
*Vital parameters*		
Heart rate (/min)^a^	451	88 ± 19 86 [76, 100]
Systolic blood pressure (mmHg)^a^	446	133 ± 22 130 [120, 145]
Respiratory rate (/min)^a^	356	20 ± 6 18 [15, 22]
spO2 (%)^a^	442	93,3 ± 5,8 95 [91, 97]
*Laboratory findings*		
Lymphocytes (/nl)	294	1.73 ± 3.48 1.00 [0.66, 1.40]
Leukocytes (/nl)^a^	478	7.9 ± 4.1 6.9 [5.1, 9.6]
Neutrophile granulocytes (/nl)	315	6,5 ± 10,9 4,8 [3.3, 7.3]
Creatinine (mg/dl)^a^	474	1,37 ± 1,59 1.0 [0.80, 1.40]
C-reactive protein (mg/l)^a^	474	84 ± 80 64 [23, 118]
Lactate dehydrogenase (U/l)^a^	358	417 ± 745 322 [252, 448]
aPTT (s)^a^	370	35 ± 16 32 [29, 36]
Potassium (mmol/l)^a^	455	4.04 ± 0.60 3.99 [3.66, 4.36]
Procalcitonin (µg/l)	321	1.32 ± 10.37 0.09 [0.05, 0.25]
pH	369	7.43 [7.40, 7.46]
pO2 (mmHg)	361	55 ± 40 50 [28, 68]
pCO2 (mmHg)	367	39 ± 9 37 [33, 43]
sO2 (mmHg)	363	54 ± 35 56 [25, 89]
*Comorbidities*		
Total number of comorbidities^a^	445	–
No comorbidity		27 (122)
1 comorbidity		22 (100)
2 comorbidities		21 (95)
3 comorbidities		15 (67)
4 comorbidities		8.8 (39)
5 comorbidities		3.4 (15)
6 comorbidities		1.6 (7)
*Type of comorbidities*		
Chronic kidney disease	468	18 (86)
Pulmonary disease	462	18 (82)
Diabetes mellitus	471	24 (112)
Dyslipidemia	451	14 (65)
Vascular/coronary artery disease	490	27 (131)
Hypertension	461	57 (265)
Cardiomyopathy	461	5.2 (24)
Tumor disease^a^	190	19 (93)
*Medication*		
Antiplatelet medication^a^	490	27(130)
RAAS-Inhibitors^a^	490	42 (204)
Antidiabetic medication^a^	490	22 (106)
Immunosuppressive medication^a^	490	18 (87)
Statins^a^	459	21 (96)
Oral anticoagulation^a^	467	14 (67)
Diuretics^a^	457	24 (110)
Proton pump inhibitors^a^	453	30 (135)

Continuous data are displayed as mean ± standard deviation/median [first quartile, third quartile], Categorical data are displayed as proportion (total number)

*BMI*, body mass index, *spO2*, saturation of peripheral oxygen, *aPTT*, activated partial thromboplastin time, *pO2*, partial pressure of oxygen, *pCO2*, partial pressure of carbon dioxide, *sO2*, saturation of oxygen, *RAAS-Inhibitors*, renin–angiotensin–aldosterone system-Inhibitors

^a^Variables included into the analysis

### Outcome definition

We focussed on the prediction of three critical in-hospital events: ICU admission, IMV support, and/or death. Because each event is of similar clinical interest and importance to guide decisions in the admission scenario, we have combined the three clinical endpoints into a single composite outcome measure.

### Model approaches

As a classical approach, a LR model on all variables was fitted and ridge regression estimators were determined. Continuous variables were transformed using restricted cubic splines with three knots (10th, 50th and 90th percentile) to take possible non-linearities into account. A fast backward approach was applied on a linear model regressing all variables on the estimated linear predictions of LR. The full model shows R2 = 1. Simplification of any degree could be applied by dropping variables. The final LR model approximates 91% of the full model, exhibits low Akaike information criteria and avoids overfitting [13, 14]. Among ML models, three regularized approaches were used that shrink parameter values by assigning a penalty on the estimates: the least absolute shrinkage and selection operator (LASSO) approach (L1, limits the sum of the absolute parameter values); the ridge regression (L2, restricts the sum of the squared parameter values; and the elastic net (EN, restricts the squared and absolute values) [15, 16]. In addition to the parametric L1, L2 and EN models, which used untransformed continuous variables, we used two ML models with the ability to also capture non-linear effects: the support vector machine [SVM] with radial function and random forest (RF). The SVM classifier projects data into a higher dimensional space and separates 2 groups by using the radial function and hyperplanes that best differentiate between hyperplane-bounded regions with the longest margin (distance) [17]. RF repeatedly splits datasets by recursive partitioning to maximize data separation, resulting in a tree-like structure of aggregated predictions, where a random sample of the features is considered in every tree split. RF model tuning is applied to the number of predictors randomly sampled at each split and the number of trees [18]. A detailed description of the modelling approaches and the algorithmic workflow for parameter estimation and model evaluation is presented in Additional file 1: Table S1.

### Model development

The following procedures were performed 50 times for each model approach: The data were randomly split for model building (training set: 75%) and model validation (test set: 25% of the data). The split was stratified by the output variable to obtain constant event rates in the subsamples. Bootstrap resampling (B = 10) was applied on the training data to determine the best tuning parameter (combination) based on the Brier score [19]. Model parameters were estimated on the training data conditional on the value(s) of the tuning parameters. Model validation is applied on the test dataset. In total numbers, 368 patients were used for training and 122 for validation.

### Performance evaluation

Predictive performance of models was assessed using area under the receiver operating characteristic curves [20] (AUC, pairs of observations with concordant ordering of predictions and true values) and the Brier score [19] (mean squared error between predictions and true outcome values). Results were summarized as boxplot and in tables as medians (inter-quartile ranges). The predictors most relevant to model predictions were determined via the loss of variable importance (1-AUC) after permutation [21]. Partial dependence profile (PDP) plots were used to visualize the relationship between predictor variables and the average model prediction [22].

### Computation

The entire analysis was carried out in R (R Core Team, 2021, Vienna, Austria) on a Linux-based system [21]. Data were processed using the tidyverse library and the Hmisc package in R [23, 24]. Models were constructed with the tidymodels package [25]. Predictor variable importance was assessed using the DALEX package [26]. Results were visualized with the ggplot2 library [27]. The detailed code snippets using R are presented in Additional file 1: Table S1.

## Results

### Patient outcomes

**Fig. 1:**
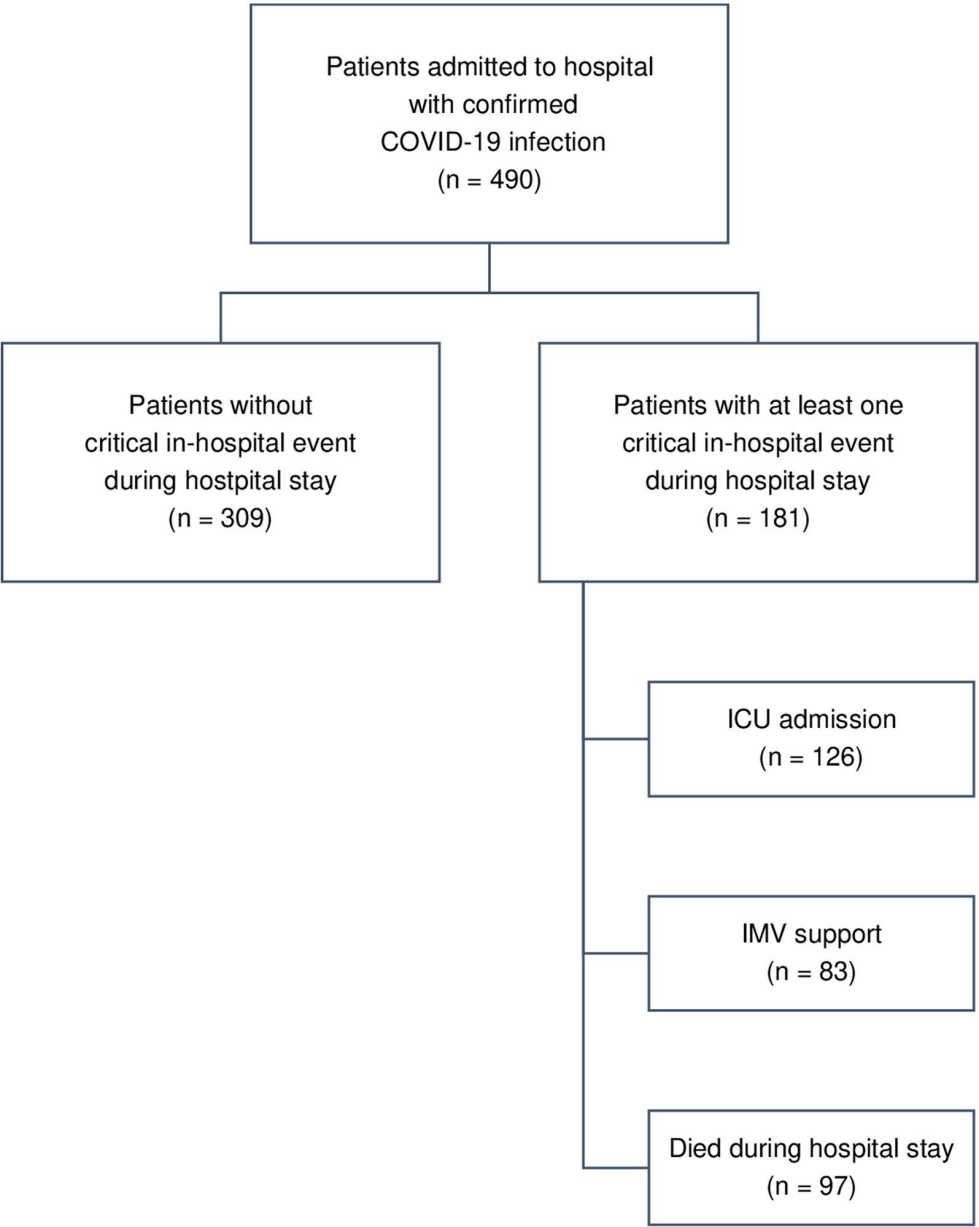
Patient pathways and outcomes. Prediction models use admission data of COVID-19-infected patient´s clinical data on hospital admission for predicting at least one of three critical in-hospital events during the remainder hospital stay

Of 490 patients who entered the hospital, 126 (26%) required transmission to ICU, 83 (17%) received IMV support, and 97 (20%) died during the remainder hospital stay (Fig. 1). The joint occurrences and combinations of these three critical events are shown in Additional file 2: Table S2. Our models use data collected during admission to predict whether a patient reached any of these three clinical end point outcomes. Overall, 181 patients (37%) had experienced at least one critical in-hospital event.

### Baseline characteristics

Table 1 shows the baseline features of the study cohort (median age: 71 years, 58% male patients) on hospital admission. Of 490 patients, 323 (72%) had at least one coexisting comorbidity, with the highest prevalence for hypertension (57%), vascular/coronary artery disease (27%) and diabetes mellitus (24%). Cough (58%), dyspnoea (57%) and fever (46%) were the most common symptoms. Enhanced C - reactive protein (CRP, 91%, cut-off 5 mg/dl) and lactate dehydrogenase (LDH, 83%, cut-off 240 U/L) were the most frequent pathologic laboratory values (Table 1).

### Model performance

**Fig. 2:**
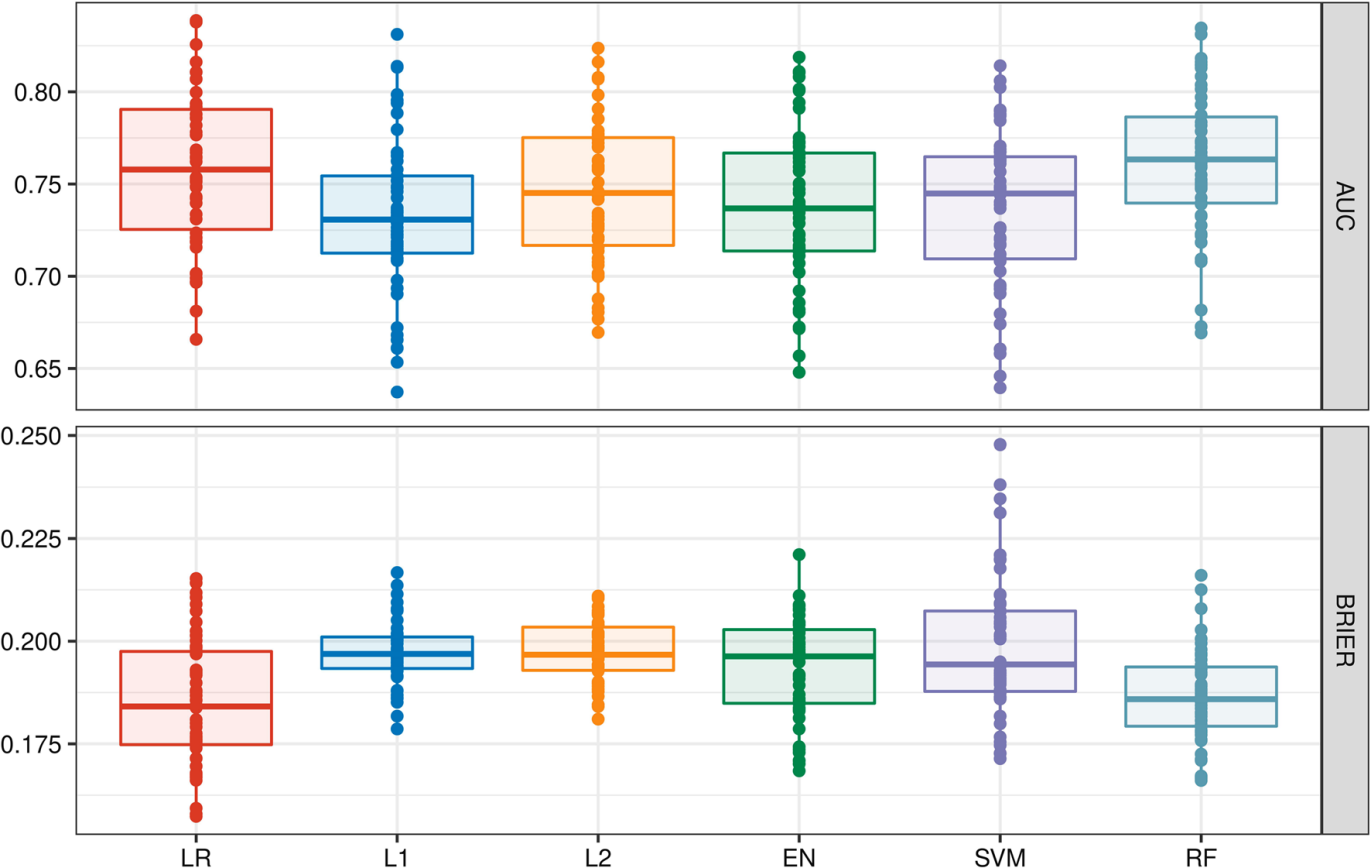
Box plots of the goodness of fit as measured by AUC values and Brier score for 50 repeatedly performed data splits for each model approach in model development for predicting critical in-hospital events using COVID-19-infected patient’s data on hospital admission. Box plots show the smallest value (low whisker), lower quartile (lower boundary of the box), median (vertical line in the box), upper quartile (upper boundary of the box), and maximum value (upper whisker)

**Fig. 3:**
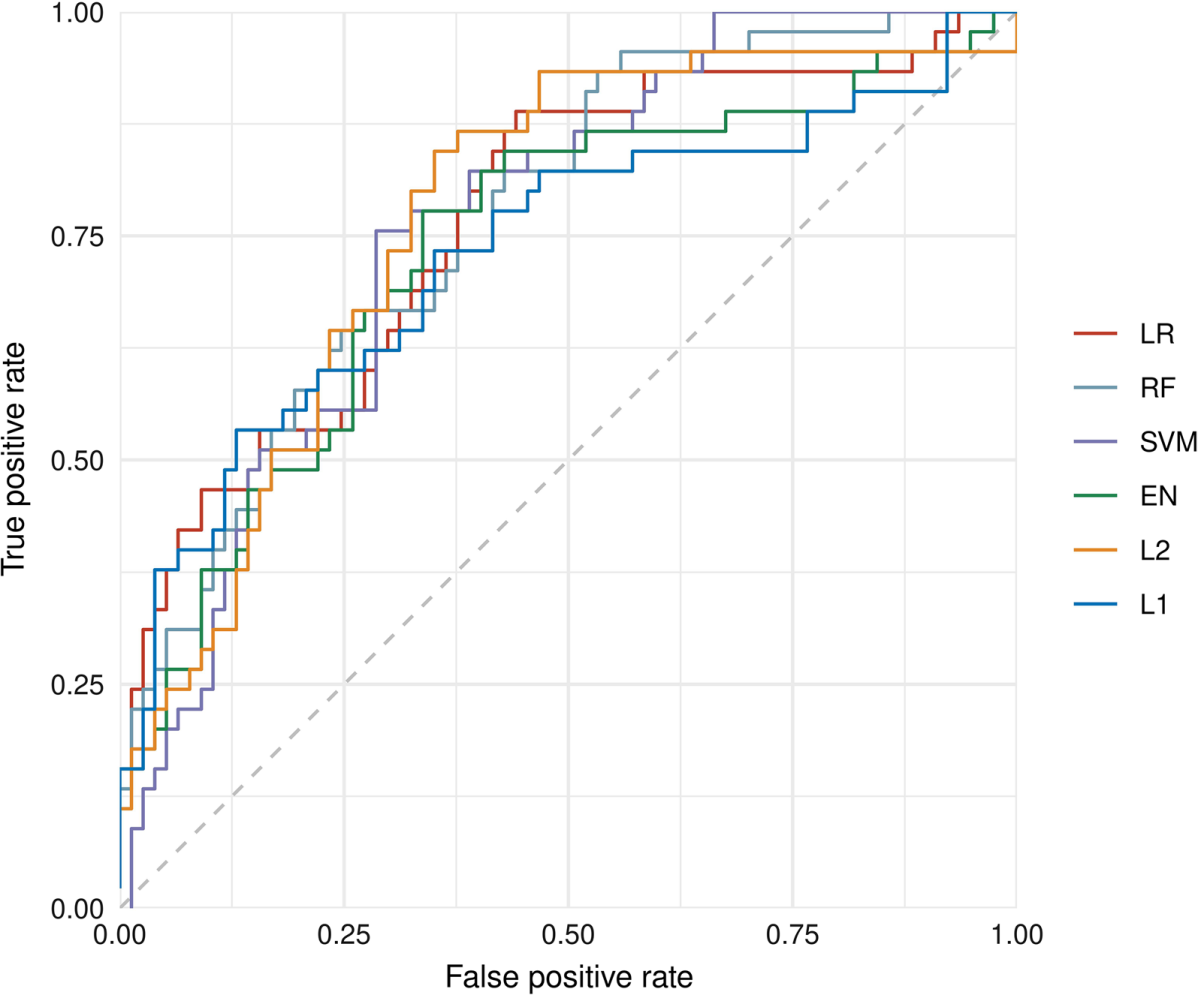
Performance comparison of model approaches for prediction of critical in-hospital events using COVID-19-infected patient’s clinical data on hospital admission displayed as ROC curves. The dashed line indicates random prediction

In general, models exhibit only small differences in performance for critical event prediction (Fig. 2). AUC values ranged from 0.731 to 0.763 (highest for the RF model) and Brier scores from 0.184 to 0.197 (lowest for the LR model) (Table 2, Fig. 3). Despite this, modest performance benefit was observed for both LR and RF over SVM (all including non-linear effects) as well as L1, L2, and EN models (all constructed with only linear effects on events) (Figs. 2 and 3, Table 2).

**Table 2:** Performance of models characterized by AUC value and Brier score for predicting critical in-hospital events using COVID-19-infected patient’s data on hospital admission

Model approach	AUC	Brier score
Logistic regression	0.758 [0.725/0.790]	0.184 [0.175/0.197]
L1 LASSO	0.731 [0.713/0.754]	0.197 [0.193/0.201]
L2 ridge regression	0.745 [0.713/0.775]	0.197 [0.193/0.203]
Elastic Net	0.737 [0.714/0.767]	0.196 [0.185/0.203]
Support vector machine	0.744 [0.710/0.765]	0.194 [0.188/0.207]
Random forest	0.763 [0.740/0.786]	0.186 [0.179/0.194]

Data are displayed as median [first quartile/third quartile]

*AUC*, Area under the curve

### Variable importance

**Fig. 4:**
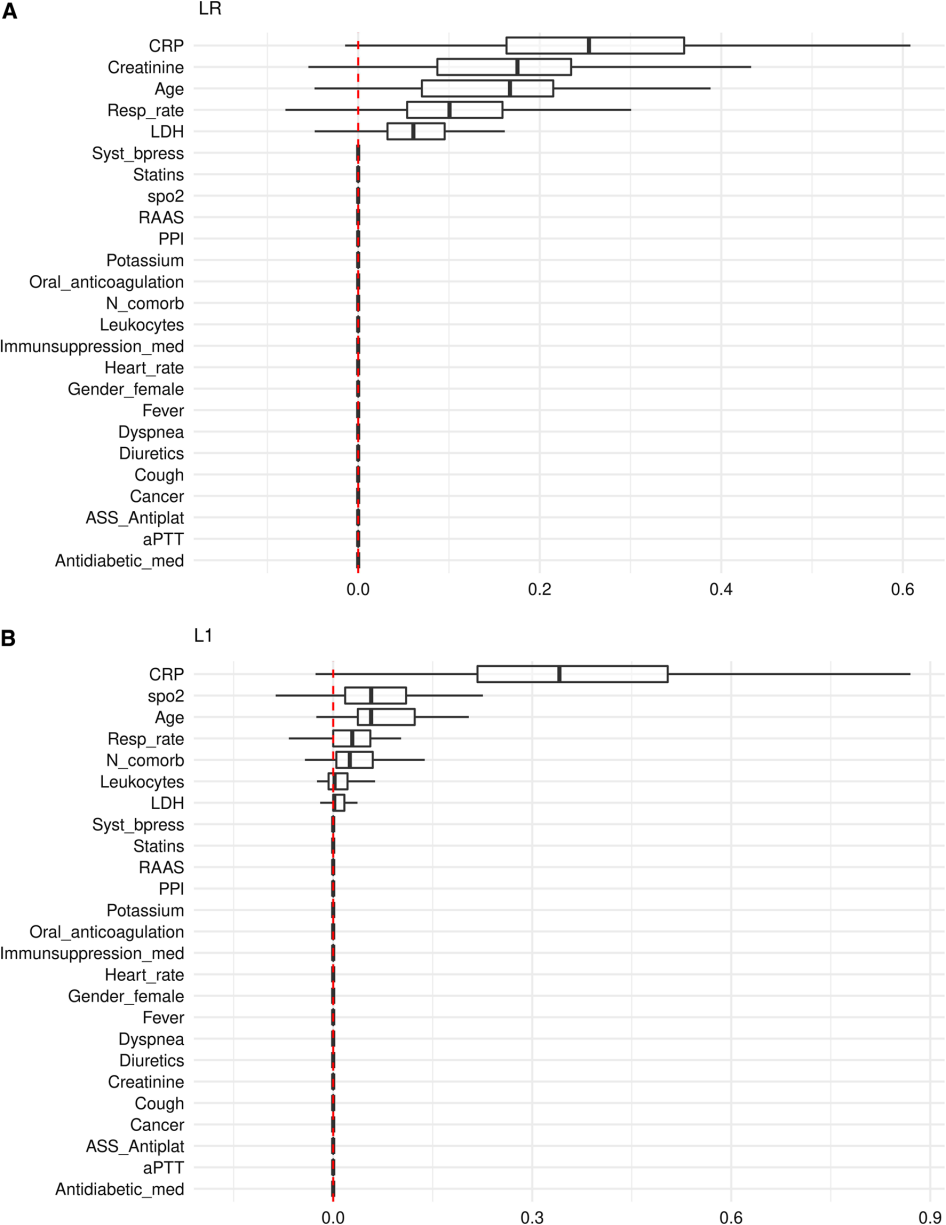

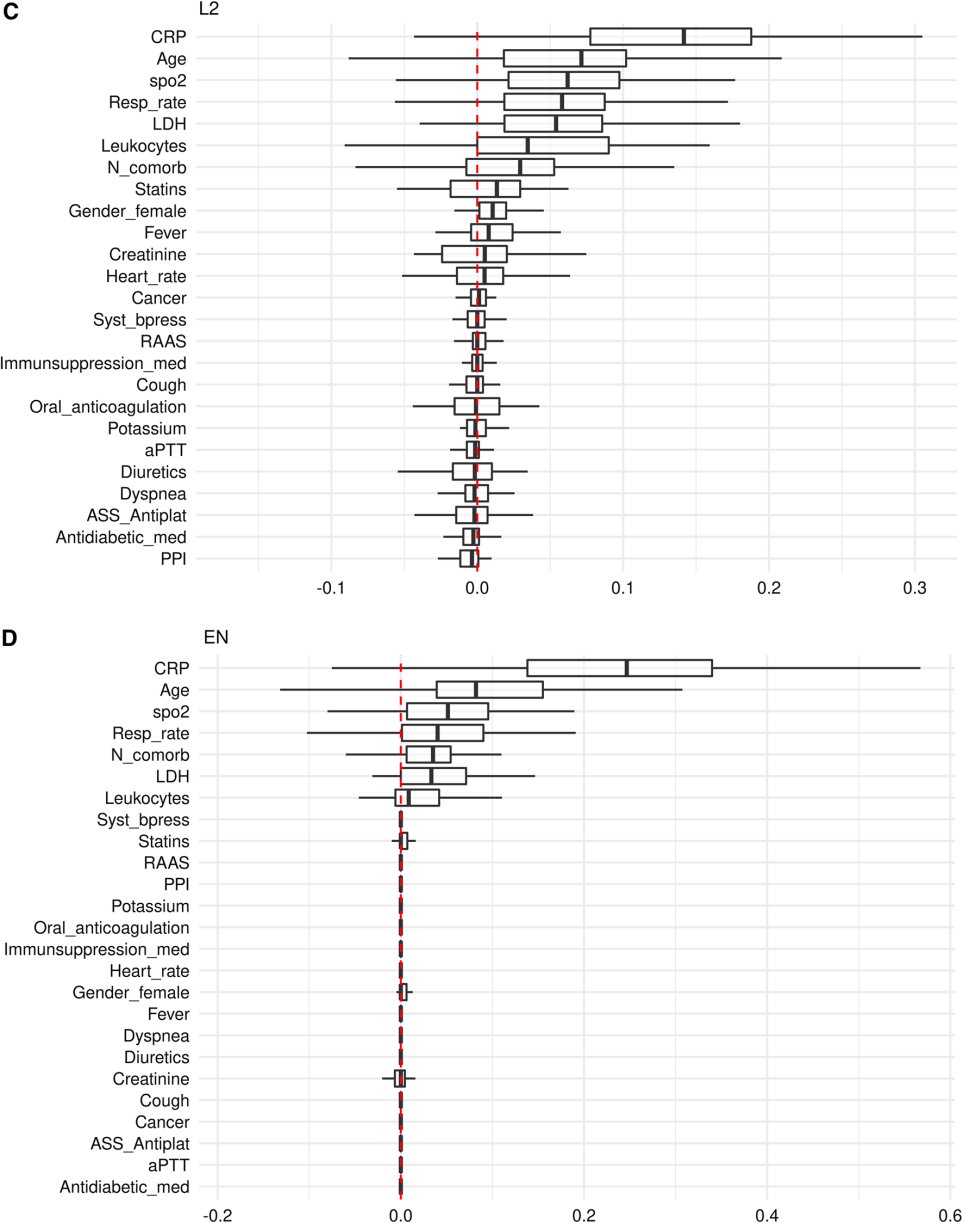

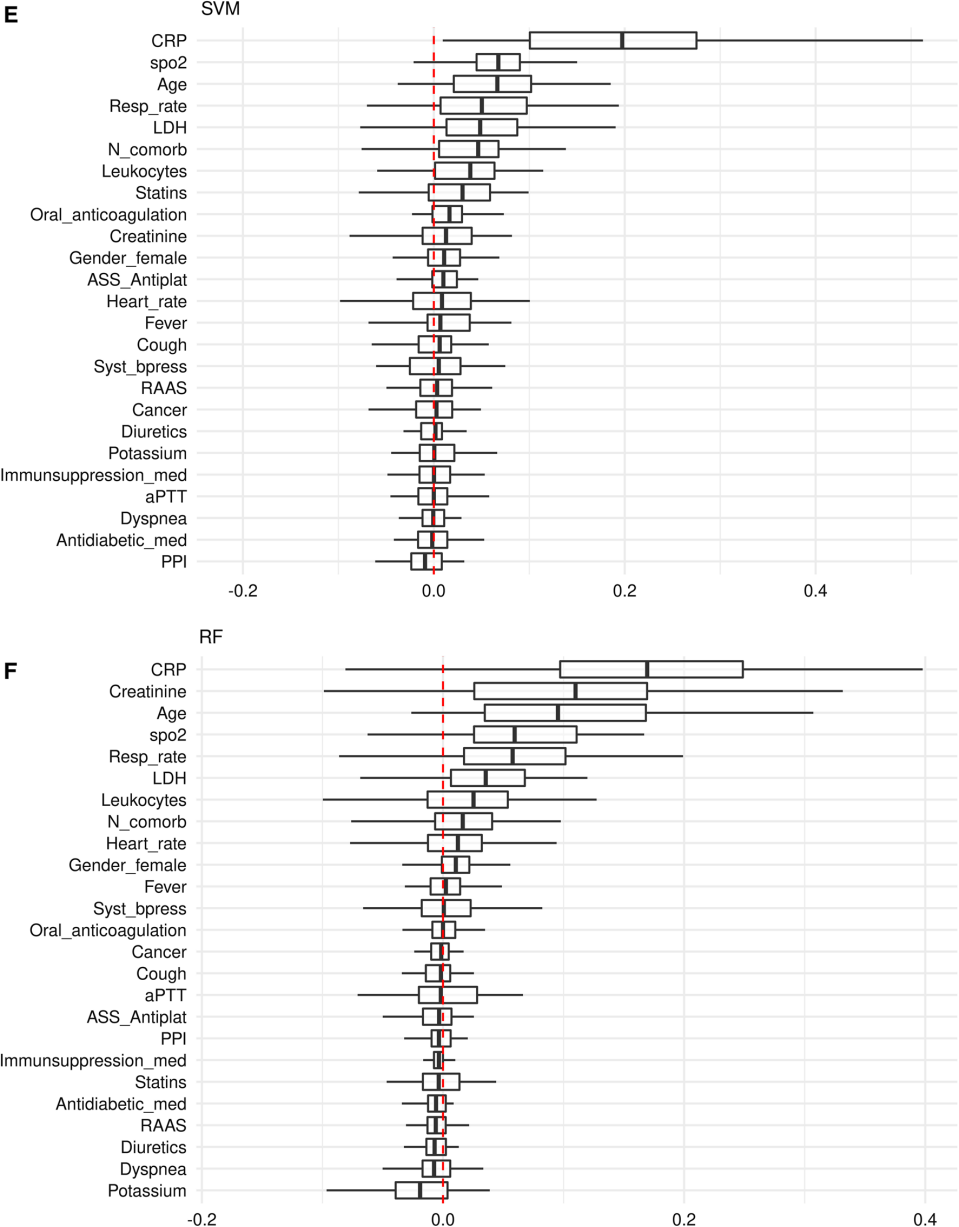
Importance of predictor variables in models predicting critical in-hospital events from COVID-19-infected patient’s clinical data on hospital admission (**A**–**F**). Permutation based performance loss of all variables for the LR model (**A**), the regularized regression models L1 (**B**), L2 (**C**) and EN (**D**), and the SVM (**E**) and RF (**F**) model

CRP obtained the highest importance across all models, and the variables age, respiratory rate and the lactate dehydrogenase (LDH) value were consistently rated among the top 7 predictor variables of the models (Figs. 4A–F, Additional file 3: Tables S3A–F). A notable difference was that oxygen saturation (spO2) was among the top 4 predictors in all ML models (Fig. 4B–F), whereas spO2 was not incorporated in the LR model (Fig. 4A). The most striking difference was observed regarding the creatinine variable, which did not play any role in the L1, L2, EN and SVM model (Fig. 4B–E). By contrast, creatinine was the 2nd most important variable for predicting critical events in the LR and RF model (Fig. 4A/F), which both slightly outperform the other models (Figs. 2 and 3, Table 2).

### Partial-dependence profiles for creatinine

**Fig. 5:**
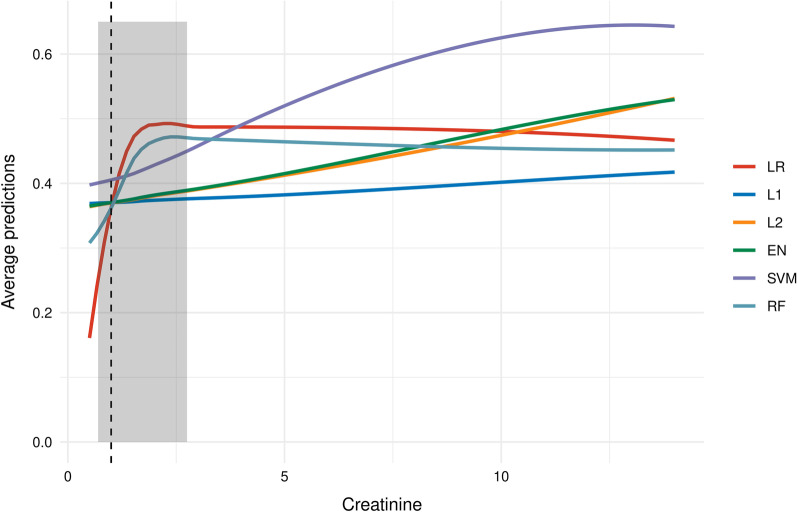
Partial-dependence profiles of creatinine for each model for predicting critical in-hospital events in COVID-19-infected patients on hospital admission. Results from 50 data splits are aggregated using local regression (LOESS). 90% of the creatinine values lie within the area represented by the grey box. The median creatinine value is represented by the vertical line. Creatinine values are given in mg/dl

Effects of creatinine on the occurrence of critical in-clinic events were revealed in LR and RF but were only marginal present in L1, L2, EN, and SVM. Ninety percent of the creatinine values ranged in the interval from 0.7 und 2.5 mg/dl. Within this interval, estimated event probabilities increased 20% in LR and RF models and ≤ 5% in all other models (Fig. 5).

### Model comparison

Both, LR and RF performed better than the regularized L1, L2 and EN models (Fig. 2, Table 2), likely due to existing non-linear effects on outcome (e.g., the creatinine level), which, due to design, are not considered in the L1, L2 and EN model (Fig. 4B–D, Additional file 3: Table S3B–D). Also, LR and RF performed also better than the SVM model (Fig. 2, Table 2), perhaps the SVM application did not sufficiently take into account the most influencing variables (e.g., creatinine), while other possibly less important variables are incorporated in the SVM model (e.g., statins, anticoagulation, and gender, Fig. 4E, Additional file 3: Table S3E). The prediction performance of the LR and RF model was almost equal. Both models consisted of similar effects and similar functional forms. The different predictors (RF has more influential variables than LR) had little additional predictive capabilities (Fig. 4A/F, Additional file 3: Table S3A/F, Fig. 5).

## Discussion

In this multicentre cohort study, we compared a classical statistical LR and five data-driven ML models to predict critical in-hospital events from COVID-19 using a 25-variable dataset from 490 admissions with 181 events. The models showed AUC means ranging from 0.731 to 0.763, indicating only small differences within an overall fair predictive performance across all models. The best performance was obtained by the RF (highest AUC value) and the LR approach (lowest Brier Score). While most top ranked influencing predictor variables were largely identical between models, specifically the variables CRP, LDH and SpO2 values and age, we also found clinically relevant differences. In particular, creatinine considerably contributed only to RF and LR.

Clinical use of ML techniques as predictive analytics is still experimental and interactions between predictors can hardly be revealed [9]. However, ML-based models predicting critical illness of COVID-19 may enable timely risk stratification of COVID-19 patients on hospital admission to personalize care and prioritize resource allocation [5, 6, 9]. While identifying actually critical ill COVID-19 patients is clinically trivial, identification of those at risk to deteriorate in advance would help to anticipate the needs for ICU and/or IMV support as well as regular beds for isolation, monitoring or best supportive end-of-life care. The rates for ICU admission (26%), IMV support (17%) and mortality (20%) in this study are in line with other studies on COVID-19 patients on hospital admission [1–4, 28–32]. For several reasons, we combined these three critical events into a single composite outcome measure. Firstly, each event is of similar clinical interest and importance to guide decisions in emergency units to early target clinical care, especially under constraint resources during peaks [6, 10]. Secondly, it avoids an arbitrary choice between critical events that refer to the same disease process [33]. Finally, it bypasses issues of competing risks, as this observational study cannot distinguish between effects of disease severity, treatment failure, resource limitations, patient wishes and non-invasive and/or invasive ventilation strategies, COVID-19 patients hospitalized for other reasons, or similarly for morbidity and mortality, whether these were related to COVID-19 and/or another underlying medical condition [34, 35].

Most top ranked predictor variables across all models examined in this study (CRP, LDH, respiratory rate and age) parallel to previously reported risk factors of critical illness in COVID-19 infection caused from the virus variant alpha (strain B.1.1.7) [5–8, 28–31, 36, 37]. Elevated CRP and LDH levels indicate inflammatory reaction and cell damage, and all four clinical parameters reflect that older patients with inflammatory COVID-19 disease and respiratory symptoms have a poor outcome [1, 6, 28–32]. Tachypnoea, rather than dyspnoea, is the first symptom of the COVID-19-related Acute Respiratory Distress Syndrome (ARDS), which is highly associated with the need for ICU and/or IMV support and/or in-hospital death [2, 3, 32]. Notably, all ML models outlined oxygen saturation as an influencing variable, whereas it was not considered in the LR approach. However, COVID-19-related ARDS is inherently an inflammatory lung disease, and the importance of CRP was ranked highest across all models. A strong linear CRP effect is confirmed by the regularized ML models (L1, L2, and EN).

Notwithstanding that the predictive benefit was small, the RF and LR model performed best in our study cohort. As the LR and RF model yielded nearly equivalent performance results, their predictive benefit likely accounts to the incorporation of non-linear effects. Notably, the SVM model performed lower, likely because SVM did not sufficiently incorporate the most influencing variables. Specifically, only the LR and RF model outlined a high non-linear effect of the creatinine level, which is consistent with studies associating kidney dysfunction in COVID-19 with morbidity and mortality [38]. Kidney involvement affects 20–40% of critically ill COVID-19 patients and can be caused by multiple pathways, including direct virologic damage of kidney cells and/or endothelial structures, hyperinflammation, cytokine release and hypercoagulability [39]. Although early recognition of kidney dysfunction in COVID-19 may limit renal failure and reduce morbidity and mortality, renal function is rarely (< 10%) reported as predictor for mortality and/or severe COVID-19 [6, 38–40]. However, this study supports that the clinical relevance of creatinine values at baseline merits further investigation in hospitalized COVID-19 patients.

Our findings may have implications for conceptualization of future ML projects. Firstly, despite of a small dataset and operations on subsamples through data splits (training/test data, tuning samples), certain purely data-driven ML methods yielded comparable performance and predictor variables similarly to a LR model. To leverage the strengths of data-driven ML technologies, future efforts should focus on augmenting the database from two directions: transversally, by including data from new sources, and longitudinally, by integrating regularly actual data from the evolving pandemic [41]. Secondly, our study outlines non-linear effects on critical events and that potentially underrated predictors such as kidney function enhance model accuracy. Thus, identifying the most influencing predictor-to-event relationships and effects is central to optimize modelling of critical event prediction, regardless of a LR or ML approach. Thirdly, predictor importance metrics, like AUC loss or PDP plots, show relative impacts of variables on prediction or how model predictions behave as a function of selected variables. Although these metrics refer to an individual ML algorithm and do not have a causal interpretation, they may help detect which predictor variables are worthy of further study [42]. Finally, a hurdle for ML application in clinical workflows is the lack of transparency and interpretability of ML models. To overcome this issue, future research may focus on selecting information from black box ML models to build interpretable but still accurate statistical glass box models with exactly determinable effects [43]. Such combinations of ML and statistical models, however, require significant knowledge in methodologies of model building and clinical expertise [44].

### Limitations

Our study has limitations. Firstly, this is an observational study from the earliest phase of the COVID-19 pandemic caused from the virus variant alpha (strain B1.1.7). Both, the nature of the study design and the limited knowledge available include methodically nonavoidable risks, such as bias in variable selection, heterogeneity of study variables and population and unstudied confounding factors. Secondly, data with informative missing and a high number of missing values were excluded. This concerned, among others, data on body mass index or D-dimers, which may offer an important contribution to prediction. Thirdly, we do not have outpatient data available. Therefore, we cannot distinguish between acute and chronic comorbidities, particularly with regard to kidney dysfunction. Fourthly, equal direction and strength of effects on different endpoints cannot be necessarily assumed. However, the composite outcome variable had valid clinical reasons. Fifthly, we compared established ML methods that are regularly applied in health care research and that were considered suitable for our dataset dimension. However, recent research extents prediction models to risk-stratify COVID-19 patients towards application of deep learning (DL) algorithms [45]. Towards a comparative performance analysis of ML and DL methods, further studies are called for. However, substantially larger datasets are required to such end, as DL methods are very data demanding and cumbersome on datasets of our dimension. Finally, the enrolled patients represent the early onset of the COVID-19 pandemic in Germany. This particularly limits generalizability to the actual pandemic period under new influences from virus mutants, [46] vaccination, [47] improved testing, [48] and evolving drug treatments, such as anticoagulation [49] or dexamethasone [50]. In addition, recent studies outline host genetic variants [51] and viral load [52] as relevant determinants of critical illness in COVID-19. However, as the pandemic and our knowledge evolve, no model will fit all regions at all times, and this study adds to the understanding of ML-based models as predictive analytics to risk-stratify COVID-19 patients into different management groups.

## Conclusion

In conclusion, we compared the performance of statistical LR and five supervised ML models for prediction of critical in-hospital events from COVID-19 using patient data at admission. Although extension of patient numbers and/or potential predictors may gain more precise estimates on diversity in performance between the models analysed, we demonstrate the superiority of models being able to investigate non-linear predictor-to-event relationships and effects, regardless of a LR or ML approach. Specifically, our data support a potentially underestimated non-linear effect of the creatinine value for indicating critical COVID-19-related patient trajectories. While our findings require external validation in larger datasets, future efforts should focus on leveraging ML technologies from static towards dynamic ML modelling solutions that learn and adapt to changes in data environments over time. This would be obtained by integrating actual data into continuous learning ML models that iteratively retrain and upgrade themselves, making them less prone to error and bias and more up-to-date for clinical decision support during actual periods of the evolving pandemic.

## Supplementary Information

**Additional file 1**: **Table S1**. Modelling approaches and algorithmic workflow for parameter estimation and model evaluation.

**Additional file 2**: **Table S2**. Distribution of patient pathways and outcomes and combinations of clinical endpoints defining critical in-hospital events in study participants (n = 490).

**Additional file 3**: **Table S3**. Variable importance for each model for prediction of critical in-hospital events using COVID-19-infected patient’s data on admission (A–F). Permutation based performance loss for the LR model (A), the regularized regression models L1 (B), L2 (C) and EN (D), and the SVM (E) and RF (F) model. The values are summarized by medians and interquartile ranges. The tables only contain predictors with median losses higher 0.

## Data Availability

All relevant data are within the manuscript and its supplementary information files. Further data are available from the corresponding author on reasonable request.

## Abbreviations

AUC: Area under the curve
COVID-19: Corona virus disease 2019
CRP: C-reactive protein
EN: Elastic net
ICU: Intensive care unit
IMV: Invasive mechanical ventilation
LASSO: Least absolute shrinkage and selection operator with logistic (L1) and ridge (L2) regression
LDH: Lactate dehydrogenase
LR: Logistic regression
ML: Machine learning
PDP: Partial dependence profile
RF: Random forest
SARS-CoV-2: Severe acute respiratory syndrome coronavirus 2
SVM: Support vector machines
