# Supplementary material for: Comparison of machine learning methods with logistic regression analysis in creating predictive models for risk of critical in-hospital events in COVID-19 patients on hospital admission
Source: BMC Med Inform Decis Mak. 2022 Nov 28;22:309. doi: 10.1186/s12911-022-02057-4 (PMC9702742; doi:10.1186/s12911-022-02057-4)
Supplement: Supplementary file 1 — Additional file 1: Table S1. Modelling approaches and algorithmic workflow for parameter estimation and model evaluation. [file 12911_2022_2057_MOESM1_ESM.docx]

| **Supplementary Table 1: Modelling approaches and algorithmic workflow for parameter estimation and model evaluation.** | | | |
| --- | --- | --- | --- |
| **No.** | **Issue** | **Explanation** | **Code snippets using R** |
| (1) | Development of the reference model (LR) | An information theory based approach to model selection was used to develop a logistic regression model aka reference model. We applied a fast backward algorithm on the saturated model and documented the loss in explained variation as well as the Akaike information criteria. The final model was chosen to be well fitted (in particular the number of variables to be included in the model) and to exhibit a high goodness of the fit (explained variation, AIC). Technically the rms::lrm function was used and continuous variables were included using spline functions rms::rcs. | # load library  library(rms)  dd <- datadist(<<dataset>>)  options(datadist = "dd")  # apply full model  f1 <- lrm(<<outcome>> ~ <<predictors>>,  data = <<dataset>>, x = TRUE, y = TRUE)  # penalization  pentrace(f1, list(simple = seq(0, 100, by = 10)))  # update shrinkage model  full_pen <- update(f1, penalty = list(simple = 30))  # model prediction  gh4$plogit <- predict(full_pen)  # fast backward method  f2 <- ols(plogit ~ <<predictors>>,  data = <<dataset>>, sigma = 1)  fastbw(f2, aics = 1e10) |
| (2) | Penalized regression (L1, L2, elastic net [EN]) | Penalized regression regression was applied via the glmnet::glmnet function. Depending on the type of penalization, the sum of absolute parameter values, the sum of quadratic parameter values or both are restricted. Regularization means that high parameter values were attributed to high influential variables and low values were assigned to variables with a low impact on the outcome. The tuning parameters (upper limit of the sum of absolute / quadratic parameter values) were estimated via bootstrapping on the training data. | # Regularized logistic regression models  # recipe  p_rec <- recipe(<<outcome>> ~ ., data = <<dataset>>) %>% step_normalize(all_numeric(), -all_outcomes())  # workflow  wf <- workflow() %>% add_recipe(p_rec)  # set up  enet_spec <- logistic_reg(penalty = tune(), mixture = tune()) %>% set_engine("glmnet")  rid_spec <- logistic_reg(penalty = tune(), mixture = 0) %>% set_engine("glmnet")  las_spec <- logistic_reg(penalty = tune(), mixture = 1) %>% set_engine("glmnet")  # model tuning  set.seed(2020)  enet_grid <- tune_grid(wf %>% add_model(enet_spec), resamples = p_boot)  rid_grid <- tune_grid(wf %>% add_model(rid_spec), resamples = p_boot)  las_grid <- tune_grid(wf %>% add_model(las_spec), resamples = p_boot)  # select best parameters  enet_best <- enet_grid %>% select_best("roc_auc")  rid_best <- rid_grid %>% select_best("roc_auc")  las_best <- las_grid %>% select_best("roc_auc") |
| (3) | Random forest (RF) | The random forest algorithm (randomForest::randomForest) was used to develop a random forest model. The model approach is based on thousands of decision trees tuned on the number of variables randomly sampled as candidates at each split and the number of trees to grow. A grid search algorithm is used. Random forest models are created on bootstrap samples. The probabilities of severe courses are derived via bagging. | # RANDOM FOREST  # set up  rf_spec <- rand_forest(mtry = tune(), trees = tune()) %>% set_mode("classification") %>% set_engine("randomForest")  # tune  rf_grid <- tune_grid(wf %>% add_model(rf_spec), resamples = p_boot)  # select best parameters  rf_best <- rf_grid %>% select_best("roc_auc") |
| (4) | Support Vector Machines (SVM) | The support vector machine classification algorithm tries to linearly separate observations with and without severe courses. The split is applied using a maximal margin between the observations. Using soft margins the costs of misclassificiations (observations within separating hyperplane) are a variable parameter in the model. A radial basis function kernel (RBF) is used to map the observations to higher dimensions to get the classification problem linearly separable (decision boundary). RBF is based on squared Euclidian distances between observations, whereas a second parameter defines the influence of an observation. | # SUPPORT VECTOR MACHINES  # set up  svm_spec <- svm_rbf(cost = tune(), rbf_sigma = tune()) %>% set_mode("classification") %>% set_engine("kernlab")  # tune  svm_grid <- tune_grid(wf %>% add_model(svm_spec), resamples = p_boot)  # select best parameters  svm_best <- svm_grid %>% select_best("roc_auc") |
| (5) | Workflow for developing the prediction rule | Algorithms of the machine learning algorithms were not called directly but within a tidymodels workflow including the normalization of the data, the tuning parameter calculation via grid search, the tuning parameter selection based on AUC and the parameter estimation on the training data. | # Prediction rules using L1  las <- general %>%  mutate(  wf = map(tr, function(x) {  rec <- recipe(<<endpoint>> ~ ., data = <<dataset>>) %>% step_normalize(all_numeric(), -all_outcomes())  workflow() %>% add_recipe(rec)  }),  grid = pmap(list(ss, wf, bt), function(s, w, b) {  spec <- logistic_reg(penalty = tune(), mixture = 1) %>% set_engine("glmnet")  set.seed(s)  tune_grid(w %>% add_model(spec), resamples = b)  }),  best = map(grid, function(x) {  x %>% select_best("roc_auc")  }),  model = pmap(list(wf, best, tr), function(w, b, d) {  spec <- logistic_reg(penalty = tune(), mixture = 1) %>% set_engine("glmnet")  finalize_workflow(w %>% add_model(spec), b) %>% fit(data = d)  })) |
| (6) | Workflow for calculation of performance metrics | Model evaluation was performed on the test data including model performance (DALEX::model_performance), effects (DALEX:: model_parts with the loss_one_minus_auc argument) and functional form of the continuous predictors (DALEX::model_profile). | # Performance metrics  all <- <<all_models>> %>%  mutate(explain = map2(model, ts, function(m, d) {  explain_tidymodels(m, data = d, y = d$vio == "yes", verbose = FALSE)  })) %>%  mutate(perform = map(explain, model_performance),  auc = map(perform, function(x) x$measures$auc),  brier = map(perform, function(x) mean(x$residuals$diff^2, na.rm = TRUE))) %>%  mutate(vip = map2(ss, explain, function(s, e) {  set.seed(s)  model_parts(explainer = e, loss_function = loss_one_minus_auc, B = 1)  })) %>%  mutate(vipt = map(vip, function(x) {  as_tibble(x) %>%  mutate(ref = x %>% filter(variable == "_full_model_") %>% pull(dropout_loss),  rel = (dropout_loss - ref) / ref) %>%  filter(!variable %in% c("_baseline_", "_full_model_")) %>%  mutate(variable = as.character(variable)) %>%  dplyr::select(variable, rel)  }),  pd_crea = map(explain, function(x) {  crea <- model_profile(explainer = x, variables = "Creatinine")  crea$agr_profiles %>% as_tibble() %>%  dplyr::select(-5) %>%  set_names(c("variable", "l", "x", "yhat"))  })) |

**Supplementary Table 1.**Detailed algorithmic and technical workflow used for the modelling approaches, parameter estimation and model evaluation.
