# Supplementary material for: Comparison of machine learning methods with logistic regression analysis in creating predictive models for risk of critical in-hospital events in COVID-19 patients on hospital admission
Source: BMC Med Inform Decis Mak. 2022 Nov 28;22:309. doi: 10.1186/s12911-022-02057-4 (PMC9702742; doi:10.1186/s12911-022-02057-4)
Supplement: Supplementary file 2 — Additional file 2: Table S2. Distribution of patient pathways and outcomes and combinations of clinical endpoints defining critical in-hospital events in study participants (n = 490). [file 12911_2022_2057_MOESM2_ESM.docx]

| Supplementary Table 2: Distribution of patient pathways and outcomes and combinations of clinical endpoints defining critical in-hospital events in study participants (n = 490) | |
| --- | --- |
| Clinical endpoints | Proportion of  study participants  Number (%) |
| Alive^1^  without IMV/ICU | 309 (63.0) |
| Alive + IMV | 2 (0.4) |
| Alive + ICU | 40 (8,2) |
| Alive + ICU + IMV | 42 (8,6) |
| Dead^2^ | 50 (10.2) |
| Dead + IMV | 3 (0,6) |
| Dead + ICU | 8 (1.6) |
| Dead + ICU + IMV | 36 (7,3) |
| ≥1 Critical in-hospital event^3^ | 181 (37.0) |
| Abbreviations: ICU, intensive care unit, IMV, invasive mechanical ventilation;  ^1^discharge alive from hospital; ^2^death during hospital stay; ^3^death and/or ICU transfer and/or IMV support | |

**Supplementary Table 2.**Joint occurrence of critical in-hospital events in COVID-19-infected patients during the hospital stay.
