# Supplementary material for: Comparison of machine learning methods with logistic regression analysis in creating predictive models for risk of critical in-hospital events in COVID-19 patients on hospital admission
Source: BMC Med Inform Decis Mak. 2022 Nov 28;22:309. doi: 10.1186/s12911-022-02057-4 (PMC9702742; doi:10.1186/s12911-022-02057-4)
Supplement: Supplementary file 3 — Additional file 3: Table S3. Variable importance for each model for prediction of critical in-hospital events using COVID-19-infected patient’s data on admission (A–F). Permutation based performance loss for the LR model (A), the regularized regression models L1 (B), L2 (C) and EN (D), and the SVM (E) and RF (F) model. The values are summarized by medians and interquartile ranges. The tables only contain predictors with median losses higher 0. [file 12911_2022_2057_MOESM3_ESM.docx]

| **Supplementary Table 3A: Variable importance table of the Logistic Regression model:** | |
| --- | --- |
| Variable | 1-AUC Loss after permutations |
| C-reactive protein | 0,254 |
| Creatinine | 0,175 |
| Age | 0,167 |
| Respiratory rate | 0,100 |
| Lactate dehydrogenase | 0,061 |
| 1-AUC Loss after permutations is given as Median | |

| **Supplementary Table 3B: Variable importance table of the L1 LASSO model:** | |
| --- | --- |
| Variable | 1-AUC Loss after permutations |
| C-reactive protein | 0,341 |
| spO2 | 0,057 |
| Age | 0,057 |
| Respiratory rate | 0,029 |
| Number of comorbidities | 0,025 |
| Leukocytes | 0,002 |
| Lactate dehydrogenase | 0,002 |
| Abbreviations: spO2: saturation of peripheral oxygen  1-AUC Loss after permutations is given as Median | |

| **Supplementary Table 3C: Variable importance table of the L2 Ridge regression model:** | |
| --- | --- |
| Variable | 1-AUC Loss after permutations |
| C-reactive protein | 0.141 |
| Age | 0.071 |
| spO2 | 0.061 |
| Respiratory rate | 0.058 |
| Lactate dehydrogenase | 0.054 |
| Leukocytes | 0.034 |
| Number of comorbidities | 0.029 |
| Statins | 0.013 |
| Gender female | 0.010 |
| Fever | 0.007 |
| Creatinine | 0.005 |
| Heart rate | 0.004 |
| Cancer | 0.001 |
| Abbreviations: spO2: saturation of peripheral oxygen  1-AUC Loss after permutations is given as Median | |

| **Supplementary Table 3D: Variable importance table of the Elastic net model:** | |
| --- | --- |
| Variable | 1-AUC Loss after permutations |
| C-reactive protein | 0.246 |
| Age | 0.082 |
| spO2 | 0.051 |
| Respiratory Rate | 0.040 |
| Number of comorbidities | 0.035 |
| Lactate dehydrogenase | 0.033 |
| Leukocytes | 0.008 |
| Abbreviations: spO2: saturation of peripheral oxygen  1-AUC Loss after permutations is given as Median | |

| **Supplementary Table 3E: Variable importance table of the Support Vector Machine model:** | |
| --- | --- |
| Variable | 1-AUC Loss after permutations |
| C-reactive protein | 0.197 |
| spO2 | 0.067 |
| Age | 0.066 |
| Respiratory rate | 0.050 |
| Lactate dehydrogenase | 0.048 |
| Number of comorbidities | 0.046 |
| Leukocytes | 0.038 |
| Statins | 0.030 |
| Anticoagulation | 0.016 |
| Creatinine | 0.012 |
| Gender female | 0.010 |
| Antiplatelet therapy | 0.010 |
| Heart rate | 0.008 |
| Fever | 0.006 |
| Cough | 0.006 |
| Systolic blood pressure | 0.005 |
| RAAS-Inhibitors | 0.003 |
| Cancer | 0.003 |
| Diuretics | 0.002 |
| Potassium | 0.001 |
| Immunosuppressive medication | 0.001 |
| Abbreviations: spO2: saturation of peripheral oxygen, RAAS-Inhibitors: renin-angiotension-aldosterone-system-Inhibitors  1-AUC Loss after permutations is given as Median | |

| **Supplementary Table 3F: Variable importance table of the Random Forest model:** | |
| --- | --- |
| Variable | 1-AUC Loss after permutations |
| C-reactive protein | 0.169 |
| Creatinine | 0.109 |
| Age | 0.095 |
| spO2 | 0.059 |
| Respiratory rate | 0.057 |
| Lactate dehydrogenase | 0.035 |
| Leukocytes | 0.025 |
| Number of comorbidities | 0.016 |
| Heart rate | 0.012 |
| Gender female | 0.010 |
| Fever | 0.002 |
| Systolic blood pressure | 0.001 |
| Abbreviations: spO2: saturation of peripheral oxygen  1-AUC Loss after permutations is given as Median | |

**Supplementary Table 3.** Variable importance for each model for prediction of critical in-hospital events using COVID-19-infected patient’s data on admission (**A-F**). Permutation based performance loss for the LR model (**A**), the regularized regression models L1 (**B**), L2 (**C**) and EN (**D**), and the SVM (**E**) and RF (**F**) model. The values are summarized by medians and interquartile ranges. The tables only contain predictors with median losses higher 0.
